# Comparative analysis of clinical endpoint progression rates in sporadic Alzheimer’s disease and Down Syndrome‐related Alzheimer’s disease

**DOI:** 10.1002/alz.090479

**Published:** 2025-01-09

**Authors:** Michael Rafii, Jessie Nicodemus‐Johnson, Suzanne B. Hendrix, Jonathan Wagg, Nicolas Fournier, Olivier Sol, Saskia Delpretti, Julien Rongere, Claude Rudaz‐Kohler, Marie Kosco‐Vilbois, Andrea Pfeifer, Johannes Streffer, Nuno Mendonca, Mitchell Wassom, Zayne Kirkham

**Affiliations:** ^1^ Alzheimer’s Therapeutic Research Institute, University of Southern California, San Diego, CA USA; ^2^ Pentara Corporation, Salt Lake City, UT USA; ^3^ AC Immune SA, Lausanne Switzerland; ^4^ AC Immune, Lousanne Switzerland; ^5^ AC Immune SA, Lousanne Switzerland; ^6^ AC Immune SA, Lausanne, Vaud Switzerland; ^7^ Reference Center for Biological Markers of Dementia (BIODEM), University of Antwerp, Antwerp Belgium; ^8^ Pentara, Salt Lake CIty, UT USA

## Abstract

**Background:**

Clinical assessments utilized in trials to measure progression in sporadic Alzheimer’s disease (SAD) such as the MMSE, ADAS‐Cog, CDR‐SB and ADCS‐ADL are well established. Distinct assessments are utilized for Down Syndrome‐related Alzheimer’s disease (DSAD). Although these assessments are less well established, they probe comparable domains of cognition and function. We quantitatively compared progression rates for SAD assessments with comparable DSAD composite assessments and explored their utility in clinical trials.

**Methods:**

The MMSE, ADAS‐Cog, CDR‐SB and ADCS‐ADL were mapped to comparable DSAD composite endpoints. Longitudinal data were sourced from the ADNI (SAD: n = 464) and ABC‐DS (DSAD: n = 393). Clinical endpoint progression rates were quantified as Mean to Standard Deviation Ratios (MSDR) of change across a unit time and derived using a model‐based approach. MSDRs were compared across populations and assessments. Clinical trial sample sizes were estimated to detect treatment effects using DSAD assessments.

**Results:**

Sensitivity of progression rate (MSDR) to baseline Amyloid PET was apparent for many DSAD composite measures. Sensitivity to progression for DSAD composite endpoints in the total ABC‐DS population at 18 months for MMSE, ADAS‐Cog, CDR‐SB and ADCS‐ADL were ‐0.32, ‐0.29, ‐0.40, and ‐0.22, respectively. Individuals with higher baseline Amyloid levels had increased sensitivity to progression with MSDRs at 18 months of ‐0.45, ‐0.33, ‐0.49, and ‐0.36. Power calculations of the most progressive measure in the total ABC‐DS population at 18 months (CDR‐SB DS composite; MSDR 0.4) indicate that assuming a 1:1 active: placebo ratio, 60% slowing and a dropout rate of 20%, the total sample size needed to reach an alpha of 0.05 at 80% power would be 548 subjects. Sample sizes for detecting treatment effects were reduced markedly with higher baseline Amyloid; CDR‐SB DS composite MSDR ‐0.49 requires 352 and 200 individuals total assuming 60 and 80% slowing over 18 months, respectively.

**Conclusions:**

Converging evidence demonstrates that DSAD progresses similarly to SAD. Here, we observe similar progression on composites of cognition and function between SAD and DSAD. Importantly, significantly smaller sample sizes will be required to detect treatment effects in DSAD populations when enriched for higher baseline Amyloid PET.

